# (*R*)-*N*-[(*R*)-2,2-Di­chloro-1-phenyl-2-(phenyl­sulfon­yl)eth­yl]-2-methyl­propane-2-sulfinamide

**DOI:** 10.1107/S1600536813034909

**Published:** 2014-01-08

**Authors:** Haiji Huang, Ya Li, Jingming Chu

**Affiliations:** a College of Chemistry and Chemical Engineering, Shanghai University of Engineering Science, 333 Longteng Road, Shanghai, People’s Republic of China

## Abstract

The title mol­ecule, C_18_H_21_Cl_2_NO_3_S_2_, contains one chiral carbon center and the absolute sterochemistry has been confirmed as as *R*. An intra­molecular N—H⋯O hydrogen bond occurs and the dihedral angle between the benzene rings is 64.5 (1)°. In the crystal, the mol­ecules are linked by weak C—H⋯O hydrogen bonds, forming a zigzag chain structure extending along the *c-*axis direction.

## Related literature   

For the use of β-amino sulfones as enzyme inhibitors, see: Tamamura *et al.* (2003 ▶); Nakatani *et al.* (2008 ▶); Raja *et al.* (2009 ▶). For their use as synthetic inter­mediates, see: Pauly *et al.* (1994 ▶); de Blas *et al.* (1994 ▶); Carretero *et al.* (1997 ▶); Alonso *et al.* (1997 ▶). For their synthesis, see: Zhang *et al.* (2011 ▶). For the fluorinated analogue, see: Li & Hu (2005 ▶); Liu & Hu (2010 ▶).
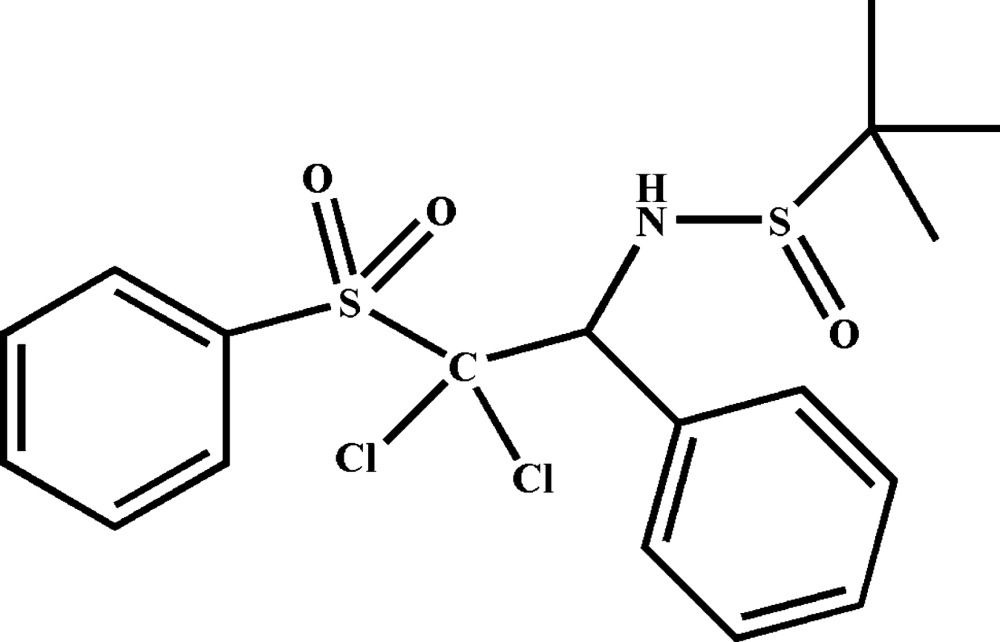


## Experimental   

### 

#### Crystal data  

C_18_H_21_Cl_2_NO_3_S_2_
*M* *_r_* = 434.38
Orthorhombic, 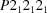

*a* = 7.924 (2) Å
*b* = 14.772 (4) Å
*c* = 18.151 (5) Å
*V* = 2124.6 (10) Å^3^
*Z* = 4
Mo *K*α radiation
μ = 0.52 mm^−1^
*T* = 293 K
0.39 × 0.30 × 0.26 mm

#### Data collection  

Bruker CCD area-detector diffractometer
Absorption correction: multi-scan (*SADABS*; Sheldrick, 2004 ▶) *T*_min_ = 0.743, *T*_max_ = 1.000
11625 measured reflections
4174 independent reflections
3834 reflections with *I* > 2σ(*I*)
*R*_int_ = 0.075

#### Refinement  

*R*[*F* ^2^ > 2σ(*F* ^2^)] = 0.038
*wR*(*F* ^2^) = 0.086
*S* = 0.99
4174 reflections
242 parameters
H atoms treated by a mixture of independent and constrained refinement
Δρ_max_ = 0.28 e Å^−3^
Δρ_min_ = −0.20 e Å^−3^
Absolute structure: Flack, 1983 ▶: 1786 Friedel pairs
Absolute structure parameter: −0.04 (6)

### 

Data collection: *SMART* (Bruker, 2007 ▶); cell refinement: *SAINT* (Bruker, 2007 ▶); data reduction: *SAINT*; program(s) used to solve structure: *SHELXS97* (Sheldrick, 2008 ▶); program(s) used to refine structure: *SHELXL97* (Sheldrick, 2008 ▶); molecular graphics: *SHELXTL* (Sheldrick, 2008 ▶); software used to prepare material for publication: *SHELXTL*.

## Supplementary Material

Crystal structure: contains datablock(s) I. DOI: 10.1107/S1600536813034909/zs2283sup1.cif

Structure factors: contains datablock(s) I. DOI: 10.1107/S1600536813034909/zs2283Isup2.hkl

Supporting information file. DOI: 10.1107/S1600536813034909/zs2283Isup3.cml

CCDC reference:

Additional supporting information:  crystallographic information; 3D view; checkCIF report

## Figures and Tables

**Table 1:** Hydrogen-bond geometry (Å, °)

*D*—H⋯*A*	*D*—H	H⋯*A*	*D*⋯*A*	*D*—H⋯*A*
C17—H17⋯O1^i^	0.93	2.44	3.236 (4)	144
N1—H1⋯O2	0.81 (3)	2.19 (3)	2.889 (3)	145 (3)

Symmetry code: (i) 
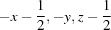
.

**Fig. 1.**
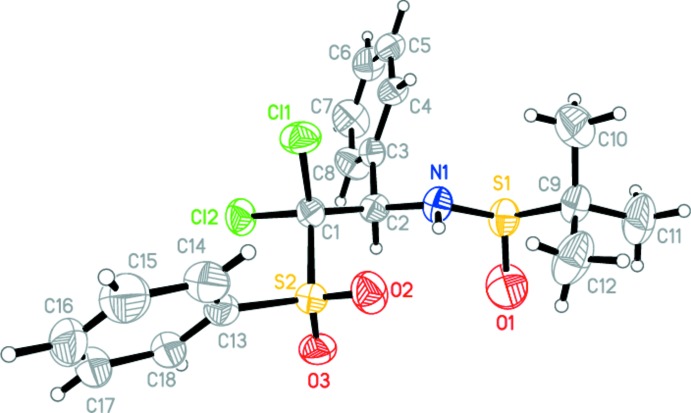
Molecular structure of the title compound. The displacement ellipsoids are drawn at the 50% probability level. H-atoms are shown by small circles of arbitrary radii.

**Fig. 2.**
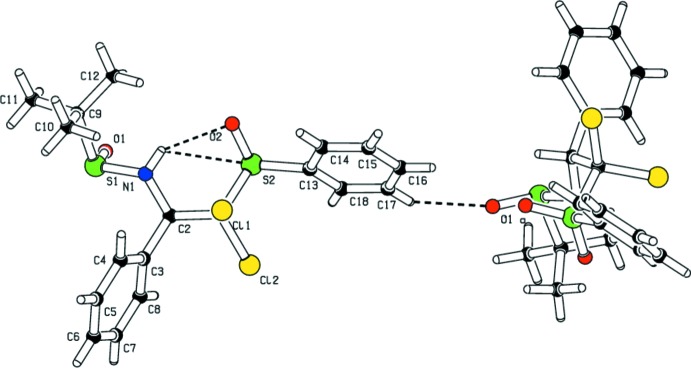
Intramolecular N—H···O and intermolecular C—H···O interactions in the title compound, shown as dashed lines. For symmetry code a: -x - 1/2, -y, z - 1/2.

**Fig. 3.**
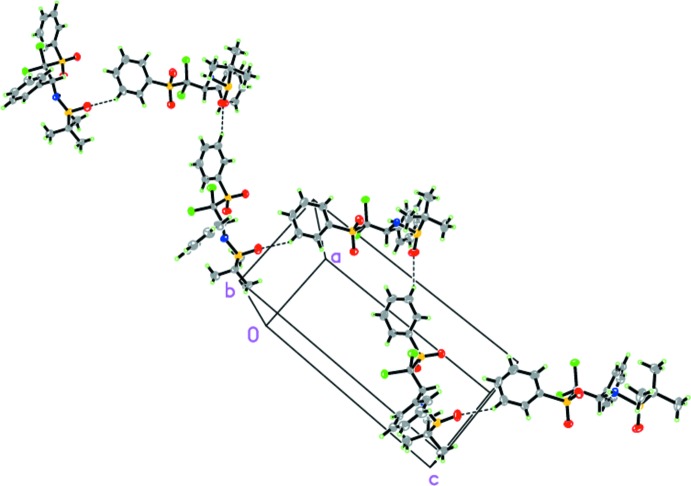
The zigzag chain formed via intermolecular C—H···O interactions, viewed along the b axis. The intramolecular hydrogen bonds are omitted for clarity.

## supplementary crystallographic information

### 1. Comment

The β-amino sulfone motif is of significant importance because it was found to play important roles in a variety of bioactive compounds (Tamamura *et al.*, 2003; Nakatani *et al.*, 2008). Besides, β-amino sulfones are valuable intermediates in the preparation of a wide variety of interesting compounds (Pauly *et al.*, 1994; Zhang *et al.*, 2011). Herein, we report the crystal structure of a α,α-dichloro-β-amino sulfone, the title compound, C_18_H_21_Cl_2_NO_3_S_2_, which may find potential use in the synthesis of bioactive compounds.

In this compound (Fig. 1), the benzene rings *A* (C3–C8) and *B* (C13-C18) are planar with r. m. s. deviations of 0.0053 Å and 0.0050 Å, respectively. The dihedral angle between *A* and *B* is 64.5 (1)°. An intramolecular N1—H···O2 hydrogen-bonding interaction is present while a weak intermolecular C17—H···O1^i^ hydrogen bond (Table 1, Figs. 2,3) results in a one-dimensional zigzag chain structure extending along *c*. Present also are 40 Å^3^ solvent accessible voids. The absolute configuration at the chiral centre [C2(*R*)] has been confirmed [absolute structure parameter = -0.04 (6) for 1786 Friedel pairs] (Flack, 1983).

### 2. Experimental

Sodium bis(trimethylsilyl)amide (1.2 mmol, 1M in THF) was added slowly to a reaction mixture of (*Rs*)-*N*-benzylidene-2-methylpropane-2-sulfinamide (209 mg, 1.0 mmol) and (dichloromethylsulfonyl)benzene (225 mg, 1.0 mmol) in THF at 203 K. The mixture was stirred for 1 h at this temperature and then quenched with 1M HCl. The quenched mixture was extracted with EtOAc. After removal of the solvent, the residue was subjected to flash colume chromatography to give the title compound (226 mg: yield, 52%). The obtained compound was recrystallized from ethyl acetate/hexane (1:1) to give colorless crystals.

### 3. Refinement

All the H atoms of the phenyl groups were placed at calculated positions and treated as riding on the parent atoms, with *U*_iso_(H) = 1.2*U*_eq_(C). The methyl H atoms were placed at calculated positions and allowed to rotate, with *U*_iso_(H) = 1.5*U*_eq_(C). The H atoms of the amide group were located in a difference-Fourier map and refined isotropically. The absolute structure parameter [-0.04 (6); Flack, 1983) at the chiral center (C2) has been confirmed as *R* (1786 Friedel pairs).

### Crystal data

**Table:**

C_18_H_21_Cl_2_NO_3_S_2_	*F*(000) = 904
*M**_r_* = 434.38	*D*_x_ = 1.358 Mg m^−^^3^
Orthorhombic, *P*2_1_2_1_2_1_	Mo *K*α radiation, λ = 0.71073 Å
Hall symbol: P 2ac 2ab	Cell parameters from 4807 reflections
*a* = 7.924 (2) Å	θ = 5.3–51.2°
*b* = 14.772 (4) Å	µ = 0.52 mm^−^^1^
*c* = 18.151 (5) Å	*T* = 293 K
*V* = 2124.6 (10) Å^3^	Prismatic, colorless
*Z* = 4	0.39 × 0.30 × 0.26 mm

### Data collection

**Table:**

Bruker CCD area-detector diffractometer	4174 independent reflections
Radiation source: fine-focus sealed tube	3834 reflections with *I* > 2σ(*I*)
Graphite monochromator	*R*_int_ = 0.075
φ and ω scans	θ_max_ = 26.0°, θ_min_ = 1.8°
Absorption correction: multi-scan (*SADABS*; Sheldrick, 2004)	*h* = −9→9
*T*_min_ = 0.743, *T*_max_ = 1.000	*k* = −18→12
11625 measured reflections	*l* = −22→22

### Refinement

**Table:**

Refinement on *F*^2^	Secondary atom site location: difference Fourier map
Least-squares matrix: full	Hydrogen site location: inferred from neighbouring sites
*R*[*F*^2^ > 2σ(*F*^2^)] = 0.038	H atoms treated by a mixture of independent and constrained refinement
*wR*(*F*^2^) = 0.086	*w* = 1/[σ^2^(*F*_o_^2^) + (0.0379*P*)^2^] where *P* = (*F*_o_^2^ + 2*F*_c_^2^)/3
*S* = 0.99	(Δ/σ)_max_ < 0.001
4174 reflections	Δρ_max_ = 0.28 e Å^−^^3^
242 parameters	Δρ_min_ = −0.20 e Å^−^^3^
0 restraints	Absolute structure: Flack, 1983: 1786 Friedel pairs
Primary atom site location: structure-invariant direct methods	Absolute structure parameter: −0.04 (6)

### Special details

**Table:**

Geometry. All e.s.d.'s (except the e.s.d. in the dihedral angle between two l.s. planes) are estimated using the full covariance matrix. The cell e.s.d.'s are taken into account individually in the estimation of e.s.d.'s in distances, angles and torsion angles; correlations between e.s.d.'s in cell parameters are only used when they are defined by crystal symmetry. An approximate (isotropic) treatment of cell e.s.d.'s is used for estimating e.s.d.'s involving l.s. planes.
Refinement. Refinement of *F*^2^ against ALL reflections. The weighted *R*-factor *wR* and goodness of fit *S* are based on *F*^2^, conventional *R*-factors *R* are based on *F*, with *F* set to zero for negative *F*^2^. The threshold expression of *F*^2^ > σ(*F*^2^) is used only for calculating *R*-factors(gt) *etc*. and is not relevant to the choice of reflections for refinement. *R*-factors based on *F*^2^ are statistically about twice as large as those based on *F*, and *R*- factors based on ALL data will be even larger.

### Fractional atomic coordinates and isotropic or equivalent isotropic displacement parameters (Å^2^)

**Table:**

	*x*	*y*	*z*	*U*_iso_*/*U*_eq_	
S1	0.31897 (9)	0.05580 (5)	0.41327 (3)	0.04581 (18)	
S2	−0.02571 (8)	0.04757 (4)	0.21496 (3)	0.03700 (15)	
Cl1	0.30446 (8)	−0.00843 (5)	0.15767 (3)	0.04829 (18)	
Cl2	0.05808 (9)	−0.14312 (4)	0.19785 (4)	0.05009 (18)	
N1	0.2849 (3)	0.04579 (15)	0.32217 (10)	0.0382 (5)	
O1	0.1580 (3)	0.0660 (2)	0.45195 (11)	0.0862 (8)	
O2	0.0522 (2)	0.13428 (12)	0.22302 (11)	0.0505 (5)	
O3	−0.1488 (2)	0.01835 (13)	0.26710 (9)	0.0501 (5)	
C1	0.1468 (3)	−0.03735 (16)	0.22190 (13)	0.0356 (5)	
C2	0.2123 (3)	−0.04217 (15)	0.30187 (12)	0.0350 (5)	
H2	0.1156	−0.0537	0.3342	0.042*	
C3	0.3408 (3)	−0.11663 (16)	0.31436 (12)	0.0350 (5)	
C4	0.5097 (3)	−0.10324 (17)	0.29887 (13)	0.0392 (6)	
H4	0.5458	−0.0477	0.2805	0.047*	
C5	0.6241 (4)	−0.17113 (19)	0.31038 (14)	0.0481 (7)	
H5	0.7373	−0.1616	0.2993	0.058*	
C6	0.5737 (4)	−0.2531 (2)	0.33810 (17)	0.0584 (8)	
H6	0.6522	−0.2990	0.3456	0.070*	
C7	0.4087 (5)	−0.2670 (2)	0.3544 (2)	0.0658 (9)	
H7	0.3742	−0.3225	0.3732	0.079*	
C8	0.2916 (4)	−0.19900 (19)	0.34330 (16)	0.0534 (7)	
H8	0.1790	−0.2088	0.3554	0.064*	
C9	0.4166 (4)	0.16808 (18)	0.41012 (14)	0.0459 (7)	
C10	0.5815 (4)	0.1612 (2)	0.3691 (2)	0.0754 (10)	
H10A	0.5600	0.1451	0.3188	0.113*	
H10B	0.6509	0.1157	0.3916	0.113*	
H10C	0.6386	0.2185	0.3708	0.113*	
C11	0.4464 (5)	0.1909 (2)	0.49142 (18)	0.0764 (10)	
H11A	0.5033	0.2481	0.4951	0.115*	
H11B	0.5146	0.1446	0.5136	0.115*	
H11C	0.3399	0.1943	0.5165	0.115*	
C12	0.2961 (5)	0.2359 (2)	0.37614 (17)	0.0702 (10)	
H12A	0.3346	0.2962	0.3865	0.105*	
H12B	0.1855	0.2276	0.3966	0.105*	
H12C	0.2920	0.2270	0.3238	0.105*	
C13	−0.1098 (3)	0.04079 (18)	0.12566 (13)	0.0391 (6)	
C14	−0.0407 (4)	0.0949 (2)	0.07116 (15)	0.0514 (7)	
H14	0.0539	0.1305	0.0803	0.062*	
C15	−0.1168 (4)	0.0943 (3)	0.00274 (17)	0.0673 (9)	
H15	−0.0724	0.1292	−0.0352	0.081*	
C16	−0.2563 (4)	0.0429 (2)	−0.00949 (16)	0.0656 (9)	
H16	−0.3057	0.0432	−0.0560	0.079*	
C17	−0.3265 (4)	−0.0098 (2)	0.04547 (17)	0.0581 (8)	
H17	−0.4227	−0.0441	0.0364	0.070*	
C18	−0.2513 (3)	−0.01063 (19)	0.11397 (15)	0.0463 (6)	
H18	−0.2961	−0.0456	0.1518	0.056*	
H1	0.237 (3)	0.0892 (18)	0.3050 (14)	0.041 (8)*	

### Atomic displacement parameters (Å^2^)

**Table:**

	*U*^11^	*U*^22^	*U*^33^	*U*^12^	*U*^13^	*U*^23^
S1	0.0602 (4)	0.0465 (4)	0.0308 (3)	−0.0052 (3)	−0.0030 (3)	0.0027 (3)
S2	0.0334 (3)	0.0386 (3)	0.0389 (3)	0.0012 (3)	−0.0006 (2)	−0.0038 (3)
Cl1	0.0409 (3)	0.0641 (4)	0.0399 (3)	0.0035 (3)	0.0099 (3)	0.0063 (3)
Cl2	0.0535 (4)	0.0381 (3)	0.0587 (4)	−0.0048 (3)	−0.0079 (3)	−0.0113 (3)
N1	0.0503 (13)	0.0327 (11)	0.0316 (10)	0.0010 (11)	−0.0046 (9)	0.0017 (9)
O1	0.0792 (15)	0.126 (2)	0.0538 (12)	−0.0324 (16)	0.0265 (12)	−0.0116 (13)
O2	0.0522 (11)	0.0372 (10)	0.0621 (12)	−0.0006 (9)	−0.0135 (10)	−0.0033 (8)
O3	0.0396 (9)	0.0694 (13)	0.0412 (9)	0.0064 (9)	0.0068 (8)	−0.0025 (9)
C1	0.0339 (12)	0.0355 (13)	0.0375 (12)	−0.0035 (10)	0.0007 (10)	−0.0024 (10)
C2	0.0373 (12)	0.0343 (12)	0.0334 (11)	−0.0039 (11)	0.0056 (10)	0.0023 (10)
C3	0.0373 (13)	0.0325 (13)	0.0352 (12)	−0.0022 (10)	−0.0024 (10)	−0.0008 (10)
C4	0.0414 (14)	0.0402 (14)	0.0360 (13)	0.0010 (11)	0.0054 (11)	0.0002 (11)
C5	0.0435 (15)	0.0576 (18)	0.0433 (14)	0.0084 (13)	0.0014 (12)	−0.0091 (13)
C6	0.063 (2)	0.0442 (17)	0.0677 (19)	0.0158 (15)	−0.0101 (16)	−0.0060 (15)
C7	0.073 (2)	0.0365 (16)	0.088 (2)	−0.0026 (15)	−0.0091 (19)	0.0145 (15)
C8	0.0458 (16)	0.0450 (16)	0.0693 (18)	−0.0078 (14)	−0.0022 (15)	0.0114 (14)
C9	0.0569 (17)	0.0404 (15)	0.0403 (14)	0.0007 (13)	−0.0041 (12)	−0.0078 (11)
C10	0.064 (2)	0.077 (2)	0.085 (2)	−0.024 (2)	0.0075 (18)	−0.0120 (19)
C11	0.103 (3)	0.071 (2)	0.0547 (18)	0.001 (2)	−0.025 (2)	−0.0192 (17)
C12	0.112 (3)	0.0453 (17)	0.0533 (17)	0.0220 (19)	−0.0169 (19)	−0.0043 (14)
C13	0.0370 (13)	0.0429 (14)	0.0373 (12)	0.0088 (12)	−0.0010 (10)	−0.0008 (11)
C14	0.0466 (15)	0.0557 (17)	0.0520 (17)	0.0031 (15)	0.0024 (13)	0.0057 (13)
C15	0.071 (2)	0.087 (2)	0.0443 (17)	0.017 (2)	0.0021 (16)	0.0115 (16)
C16	0.073 (2)	0.080 (2)	0.0440 (15)	0.022 (2)	−0.0168 (15)	−0.0116 (17)
C17	0.0516 (16)	0.0634 (19)	0.0594 (17)	0.0085 (16)	−0.0146 (15)	−0.0173 (15)
C18	0.0428 (14)	0.0472 (15)	0.0489 (15)	0.0021 (13)	−0.0045 (12)	−0.0064 (12)

### Geometric parameters (Å, º)

**Table:**

S1—O1	1.464 (2)	C8—H8	0.9300
S1—N1	1.682 (2)	C9—C10	1.507 (4)
S1—C9	1.831 (3)	C9—C12	1.516 (4)
S2—O3	1.4258 (18)	C9—C11	1.532 (4)
S2—O2	1.4296 (19)	C10—H10A	0.9600
S2—C13	1.755 (2)	C10—H10B	0.9600
S2—C1	1.860 (3)	C10—H10C	0.9600
Cl1—C1	1.761 (2)	C11—H11A	0.9600
Cl2—C1	1.768 (2)	C11—H11B	0.9600
N1—C2	1.468 (3)	C11—H11C	0.9600
N1—H1	0.81 (3)	C12—H12A	0.9600
C1—C2	1.543 (3)	C12—H12B	0.9600
C2—C3	1.516 (3)	C12—H12C	0.9600
C2—H2	0.9800	C13—C18	1.371 (4)
C3—C8	1.381 (4)	C13—C14	1.385 (4)
C3—C4	1.382 (3)	C14—C15	1.381 (4)
C4—C5	1.368 (4)	C14—H14	0.9300
C4—H4	0.9300	C15—C16	1.359 (5)
C5—C6	1.371 (4)	C15—H15	0.9300
C5—H5	0.9300	C16—C17	1.382 (5)
C6—C7	1.356 (5)	C16—H16	0.9300
C6—H6	0.9300	C17—C18	1.379 (4)
C7—C8	1.382 (4)	C17—H17	0.9300
C7—H7	0.9300	C18—H18	0.9300
			
O1—S1—N1	109.91 (13)	C10—C9—C12	112.9 (3)
O1—S1—C9	106.88 (15)	C10—C9—C11	110.9 (3)
N1—S1—C9	96.71 (11)	C12—C9—C11	110.1 (3)
O3—S2—O2	119.92 (12)	C10—C9—S1	108.7 (2)
O3—S2—C13	109.64 (12)	C12—C9—S1	110.2 (2)
O2—S2—C13	108.03 (12)	C11—C9—S1	103.6 (2)
O3—S2—C1	104.70 (11)	C9—C10—H10A	109.5
O2—S2—C1	106.24 (11)	C9—C10—H10B	109.5
C13—S2—C1	107.65 (11)	H10A—C10—H10B	109.5
C2—N1—S1	112.80 (16)	C9—C10—H10C	109.5
C2—N1—H1	115.1 (19)	H10A—C10—H10C	109.5
S1—N1—H1	112.7 (19)	H10B—C10—H10C	109.5
C2—C1—Cl1	113.30 (16)	C9—C11—H11A	109.5
C2—C1—Cl2	108.97 (16)	C9—C11—H11B	109.5
Cl1—C1—Cl2	109.45 (13)	H11A—C11—H11B	109.5
C2—C1—S2	110.00 (16)	C9—C11—H11C	109.5
Cl1—C1—S2	108.24 (12)	H11A—C11—H11C	109.5
Cl2—C1—S2	106.66 (12)	H11B—C11—H11C	109.5
N1—C2—C3	110.0 (2)	C9—C12—H12A	109.5
N1—C2—C1	109.09 (18)	C9—C12—H12B	109.5
C3—C2—C1	113.58 (19)	H12A—C12—H12B	109.5
N1—C2—H2	108.0	C9—C12—H12C	109.5
C3—C2—H2	108.0	H12A—C12—H12C	109.5
C1—C2—H2	108.0	H12B—C12—H12C	109.5
C8—C3—C4	118.5 (2)	C18—C13—C14	122.2 (2)
C8—C3—C2	120.5 (2)	C18—C13—S2	119.0 (2)
C4—C3—C2	121.1 (2)	C14—C13—S2	118.5 (2)
C5—C4—C3	120.4 (2)	C15—C14—C13	117.7 (3)
C5—C4—H4	119.8	C15—C14—H14	121.1
C3—C4—H4	119.8	C13—C14—H14	121.1
C4—C5—C6	120.7 (3)	C16—C15—C14	120.4 (3)
C4—C5—H5	119.6	C16—C15—H15	119.8
C6—C5—H5	119.6	C14—C15—H15	119.8
C7—C6—C5	119.6 (3)	C15—C16—C17	121.6 (3)
C7—C6—H6	120.2	C15—C16—H16	119.2
C5—C6—H6	120.2	C17—C16—H16	119.2
C6—C7—C8	120.4 (3)	C18—C17—C16	118.8 (3)
C6—C7—H7	119.8	C18—C17—H17	120.6
C8—C7—H7	119.8	C16—C17—H17	120.6
C3—C8—C7	120.4 (3)	C13—C18—C17	119.2 (3)
C3—C8—H8	119.8	C13—C18—H18	120.4
C7—C8—H8	119.8	C17—C18—H18	120.4
			
O1—S1—N1—C2	−72.4 (2)	C4—C5—C6—C7	−0.2 (4)
C9—S1—N1—C2	176.92 (18)	C5—C6—C7—C8	0.0 (5)
O3—S2—C1—C2	−56.98 (18)	C4—C3—C8—C7	−1.8 (4)
O2—S2—C1—C2	70.87 (18)	C2—C3—C8—C7	179.8 (3)
C13—S2—C1—C2	−173.60 (16)	C6—C7—C8—C3	1.0 (5)
O3—S2—C1—Cl1	178.74 (12)	O1—S1—C9—C10	−178.7 (2)
O2—S2—C1—Cl1	−53.41 (15)	N1—S1—C9—C10	−65.5 (2)
C13—S2—C1—Cl1	62.12 (15)	O1—S1—C9—C12	−54.5 (2)
O3—S2—C1—Cl2	61.06 (14)	N1—S1—C9—C12	58.8 (2)
O2—S2—C1—Cl2	−171.09 (12)	O1—S1—C9—C11	63.3 (2)
C13—S2—C1—Cl2	−55.56 (15)	N1—S1—C9—C11	176.5 (2)
S1—N1—C2—C3	−68.1 (2)	O3—S2—C13—C18	−17.1 (2)
S1—N1—C2—C1	166.75 (16)	O2—S2—C13—C18	−149.4 (2)
Cl1—C1—C2—N1	58.3 (2)	C1—S2—C13—C18	96.3 (2)
Cl2—C1—C2—N1	−179.58 (16)	O3—S2—C13—C14	156.5 (2)
S2—C1—C2—N1	−63.0 (2)	O2—S2—C13—C14	24.2 (2)
Cl1—C1—C2—C3	−64.7 (2)	C1—S2—C13—C14	−90.2 (2)
Cl2—C1—C2—C3	57.4 (2)	C18—C13—C14—C15	−1.6 (4)
S2—C1—C2—C3	173.99 (16)	S2—C13—C14—C15	−174.9 (2)
N1—C2—C3—C8	140.1 (2)	C13—C14—C15—C16	0.9 (4)
C1—C2—C3—C8	−97.3 (3)	C14—C15—C16—C17	0.2 (5)
N1—C2—C3—C4	−38.3 (3)	C15—C16—C17—C18	−0.7 (5)
C1—C2—C3—C4	84.2 (3)	C14—C13—C18—C17	1.0 (4)
C8—C3—C4—C5	1.6 (4)	S2—C13—C18—C17	174.3 (2)
C2—C3—C4—C5	−179.9 (2)	C16—C17—C18—C13	0.1 (4)
C3—C4—C5—C6	−0.7 (4)		

### Hydrogen-bond geometry (Å, º)

**Table:**

*D*—H···*A*	*D*—H	H···*A*	*D*···*A*	*D*—H···*A*
C17—H17···O1^i^	0.93	2.44	3.236 (4)	144
N1—H1···O2	0.81 (3)	2.19 (3)	2.889 (3)	145 (3)
N1—H1···S2	0.81 (3)	2.72 (3)	3.138 (2)	114 (2)

Symmetry code: (i) −*x*−1/2, −*y*, *z*−1/2.
